# Retrospective evaluation of left ventricular eccentricity index in the assessment of precapillary pulmonary hypertension in dogs (2017–2021): 145 cases

**DOI:** 10.3389/fvets.2025.1548417

**Published:** 2025-05-21

**Authors:** Nicolas Graziano, Kris Gommeren, Annelies Valcke, Priscilla Burnotte, Dave Beeston, Tom Walker, Rebecca Gele, Marine Lekane, Anne Christine Merveille

**Affiliations:** ^1^Department of Small Animal Clinical Science, Faculty of Veterinary Medicine, University of Liège, Liège, Belgium; ^2^Willows Veterinary Centre and Referral Services, Solihull, United Kingdom; ^3^Emergency and Critical Care, BluePearl Veterinary Specialists, Sandy Springs, GA, United States

**Keywords:** echocardiography, eccentricity index, pulmonary arterial hypertension, point-of-care ultrasound (POCUS), emergency

## Abstract

**Objective:**

To determine interobserver variability of left ventricular eccentricity indices measurements in systole (EIs), diastole (EId) and at maximum flattening (EIm) by emergency and critical care residents on prerecorded cineloops in dogs with or without pulmonary hypertension. To assess whether these EI measurements allow to identify dogs with right heart changes compatible with moderate to severe pulmonary hypertension (PAH).

**Methods:**

Multicenter, retrospective, case–control study from 2017 to 2021. Medical records of dogs with stage B1 myxomatous mitral valve disease (MMVD) and dogs diagnosed with precapillary pulmonary hypertension (PCPH) via echocardiograms were reviewed. Dogs were categorized by a cardiologist into five groups (normal, B1 MMVD, mild, moderate, and severe PCPH) based on Doppler pulmonary pressure gradients and right heart morphology. Four blinded emergency and critical care residents measured EIs, EId and EIm.

**Results:**

One hundred and forty-five client-owned dogs were included. Interobserver agreement was strong, with an intraclass correlation coefficient (ICC) of 0.737 (95% CI: 0.621–0.852) across all eccentricity indices for the full study population and 0.768 (0.642–0.856) for the PAH group specifically. EIs, EId, and EIm were significantly higher in the PAH group compared to control and MMVD groups (*p* < 0.0001). The differentiation between moderate-to-severe and mild/absent PAH by EIs, EId, and EIm resulted in AUCs of 0.738, 0.834, and 0.766, with cut-off values of 1.40, 1.34, and 1.28, respectively. A gray zone approach identified 90% sensitivity for EIs (1.12), EId (1.15), and EIm (1.23), and specificity for EIs (2.27), EId (1.32), and EIm (2.1) to rule out or diagnose moderate-to-severe PAH.

**Conclusions:**

This study showed good inter-observer agreement of EIs, EIm, and EId measurement by ECC residents on prerecorded loops. EI allowed good identification of dogs with moderate to severe PAH by ECC residents.

## Introduction

Pulmonary arterial hypertension (PAH) is defined as an abnormally increased arterial pressure within the pulmonary vasculature (1). In human medicine, PAH refers to a pulmonary arterial pressure (PAP) ≥ 20 mmHg at rest, ideally measured by direct right heart catheterization (2). PAH may result from various diseases, either due to increased blood flow within the capillaries, increased pulmonary vascular resistance (precapillary PAH), and/or increased downstream resistance [postcapillary pulmonary venous hypertension (PVH)] (3). Precapillary pulmonary hypertension (PCPH) is defined as elevated pulmonary arterial pressure with a normal pulmonary arterial wedge pressure. It can arise because of various underlying conditions, such as parenchymal lung disorders, chronic hypoxia, thromboembolic events, or parasitic infections. In contrast, postcapillary pulmonary hypertension is characterized by both elevated pulmonary arterial pressure and elevated wedge pressure, typically resulting from left-sided heart disease (1–3).

PAH is a complex syndrome that often leads to changes in the right side of the heart, which can progress to right-sided heart failure and death (4). PAH can cause the interventricular septum to flatten and shift toward the left ventricle, affecting left ventricular function, a process referred to as ventricular interdependence. The flattening of the interventricular septum occurs secondary to pressure overload during systole and volume overload during diastole of the right ventricle (5–7). During systole, increased pulmonary arterial pressure leads to right ventricular pressure overload. This elevated pressure causes the septum to bow toward the left ventricle, flattening its normal curvature. In diastole, volume overload due to right ventricular dilation contributes to septal flattening, as the enlarged chamber displaces the septum even when the pressure gradient is lower (5–7). The severity of this septal flattening has been shown to be associated with outcomes in human patients with PAH (1).

EI quantifies the degree of septal flattening (7). EI is calculated as the ratio of the diameter of the left ventricle parallel to the septum to the diameter of the left ventricle perpendicular to the septum.

The EI can be calculated at different time points during the cardiac cycle, namely end-systole (EIs), end-diastole (EId) (8), and at the time of maximal septal flattening (EIm) (9, 10). To capture the maximal septal flattening associated with ventricular interdependence, EI can also be measured at the time when the left ventricle (LV) appears most compressed—that is, when the ratio of the cranio-caudal to latero-lateral LV diameter is greatest, known as EIm (9).

In pulmonary arterial hypertension (PAH), the high resistance of the pulmonary vasculature causes the right ventricle (RV) to contract for a longer period with a smaller ejection volume, impairing LV filling. This results in prolonged RV systole or isovolumic relaxation, leading to a significant delay in RV diastolic inflow compared to the LV (6). Consequently, in PAH, the RV may still be contracting while the LV is already relaxing, causing late maximal septal flattening, with EIm occurring slightly after EIs (9, 10). When there is no additional septal displacement after end-systole, EIs and EIm are (almost) equal (9).

In human medicine, although all EI measurements correlate with invasive hemodynamic parameters, EIm shows the strongest correlation (9). Furthermore, EI correlates with PAH severity and has demonstrated high inter-observer agreement, making it a convenient and reliable non-invasive tool for diagnosing and monitoring PAH (9, 11, 12).

Treatment with sildenafil is recommended for dogs presenting with moderate to severe PAH, underscoring the interest of timely recognition of this condition by emergency clinicians. Cardiac point-of-care ultrasound (POCUS), also called focused cardiac ultrasound (FOCUS), has been proposed as a rapid, bedside method for assessing PAH in dogs without advanced left sided heart disease or other right-sided cardiac conditions (13, 14, 27). A subjective scoring system, utilizing either 8 or 10 points, has been described. These scores evaluate a combination of echocardiographic signs, notably right atrial and ventricular enlargement, right ventricular hypertrophy, interventricular septal flattening, and enlargement of the pulmonary artery and trunk to calculate the composite (PHS8), as well as the presence of peritoneal effusion and/or dilation of the caudal vena cava—(PHS10) (14, 15). FOCUS assesses key aspects of cardiac function through three fundamental views, one of which is the right parasternal short axis at the level of the papillary muscle. EI, measured in this view, necessitates only a single window, as opposed to the 8- or 10-point scoring systems, which require multiple views. EI might therefore serve as a more straightforward and accessible marker of PAH for general practitioners and emergency clinicians.

Our group recently reported EI in dogs with PAH assessed by a single cardiologist (16), but the inter-observer agreement between emergency and critical care (ECC) residents and potential impact of concurrent cardiac morphological changes have not been studied in veterinary medicine. ECC residents were selected due to their role in the initial assessment of critically ill patients and for evaluation of the feasibility of EI measurements by non-cardiologist. Early identification of PAH in emergency settings may enhance patient triage and enable timely initiation of treatment, potentially leading to improved morbidity and mortality. The primary aim of this study was to assess inter-observer agreement of EIs, EId, and EIm among four small animal emergency and critical care residents in dogs with PAH, dogs with ACVIM B1 mitral valve disease without PAH, and dogs without cardiac disease. The secondary aim was to evaluate the ability of EIs, EId, and EIm to predict the presence of moderate to severe PAH.

## Materials and methods

### Population

Medical records of dogs undergoing echocardiography between 2017 and 2021 at a referral hospital and small animal teaching hospital were reviewed. Dogs were included if they had a final diagnosis of precapillary pulmonary hypertension (PCPH) confirmed by a board-certified cardiologist after full diagnostic echocardiography and had available cineloops. Dogs were excluded if they had any left- or right-sided cardiac disease other than ACVIM stage B1 myxomatous mitral valve disease (MMVD) or presumed precapillary pulmonary hypertension (PCPH). These dogs were excluded, as such modifications could have influenced EI measurements. There was no exclusions for missing data or poor image quality.

### Echocardiography

Transthoracic 2D echocardiography, M-mode echocardiography, and conventional Doppler echocardiography was performed by two board-certified veterinary cardiologists according to the recommendations of the Echocardiography Committee of the Specialty of Cardiology, American College of Veterinary Internal Medicine, using two different ultrasound units.^1^ Dogs were placed in right and left lateral recumbency, and a simultaneous one-lead echocardiogram was recorded. PAH was diagnosed and categorized retrospectively on cineloops, with cardiologists blinded to other clinical data, based on the presence of an elevated tricuspid regurgitation pressure gradient (TRPG: > 30 and < 50 mmHg for mild PAH, ≥ 50 and < 75 mmHg for moderate PAH, and ≥ 75 mmHg for severe PAH) or an elevated pulmonic regurgitation pressure gradient (PRPG: ≥ 20 and < 25 mmHg for mild PAH, ≥ 25 and < 35 mmHg for moderate PAH, and ≥ 35 mmHg for severe PAH) and indirect echocardiographic evidence of PAH. Indirect parameters were assessed at three different levels, similarly to the approach described in the ACVIM consensus statement. More specifically, pulmonary artery size (main and right branches), right ventricular size and thickness, as well as right atrial dimensions were subjectively and quantitatively evaluated in all dogs (3, 12, 17). Dogs with pulmonary arterial hypertension were included only if no left atrial dilation was present, in order to exclude postcapillary hypertension. Included dogs were divided into different groups according to Doppler echocardiographic evidence of PAH: Mild PAH group, moderate PAH group and severe PAH group. Two control groups were also included: healthy dogs with or without Doppler derived estimates of PA pressure, and dogs with ACVIM B1 MMVD and a normal pulmonary pressure gradient.

The following parameters were recorded: patient age (years), weight (kg), sex, breed, group (control, PAH, MMVD), tricuspid regurgitation pressure gradient in mmHg (TRPG), pulmonic insufficiency pressure gradient in mmHg (PRPG), and severity of the PAH.

### EI assessment

**Figure 1:**
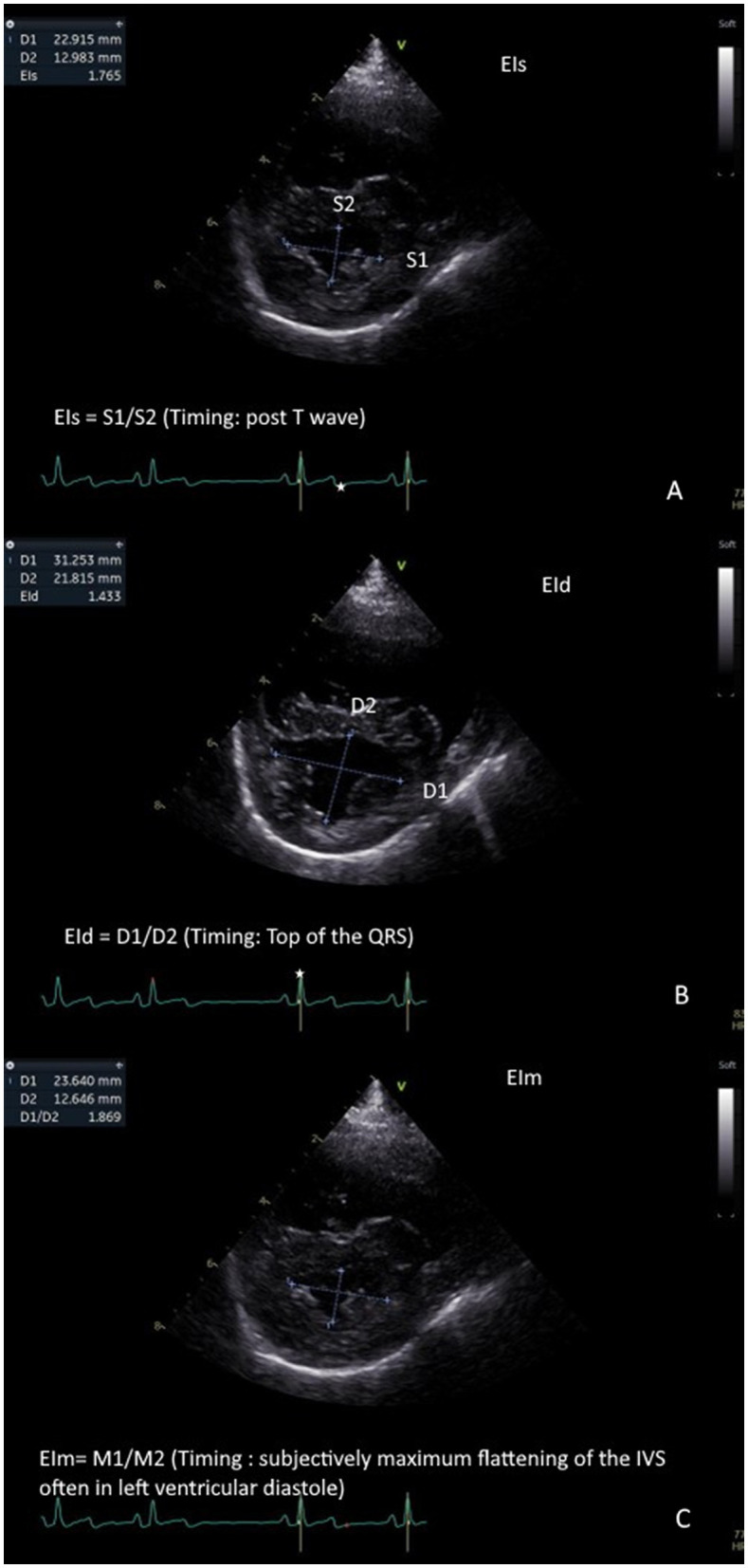
Measurement of eccentricity indices: Left ventricular eccentricity index measured at end-systole (EIs) is measured at the end of the T wave (represented as a white star on the ECG). This represents the ratio of the craniocaudal diameter of the left ventricle in systole (S1) on the latero-lateral diameter of the left ventricle in systole (S2). EIs ¼ S1/S2. **(A)**. left ventricular eccentricity index measured at end-diastole (EId) is measured at the peak of the QRS complex wave (represented as a white star on the ECG). This represents the ratio of the cranio-caudal diameter of the left ventricle in diastole (D1) on the latero-lateral diameter of the left ventricle in diastole (D2). EId ¼ D1/D2. **(B)**. Left ventricular eccentricity index measured at maximal septal flattening (EIm) is measured at the maximal interventricular septal flattening. This represents the ratio of the cranio-caudal diameter of the left ventricle (M1) on the latero-lateral diameter of the left ventricle (M2). EIm ¼ M1/M2. **(C)**.

Four small animal ECC with no prior echocardiography experience underwent a short online training course (1.5 h) on EIs, EId, and EIm assessment, delivered by a board-certified cardiologist (ACM). No formal performance assessment was conducted post-training. All residents reviewed the cineloops and were blinded to patient signalment, name, and study group (PH case vs. normal vs. B1 MMVD). Each observer individually and retrospectively performed a single measurement of EIs, EId, and EIm on each case on the transventricular right parasternal short axis cineloop at the level of the papillary muscle, as previously described by Lekane et al. (Figure 1). An electrocardiogram was used to measure EIs (at the end of the T wave) and EId (at the peak of the QRS complex), as ECG-based timing is an objective and standardized method commonly used in cardiology and provides a quality control measure to ensure consistency and reproducibility. EIm was assessed at the subjective maximal downward motion of the interventricular septum.

### Statistical methods

Inter-observer variability was assessed on four measurements for each parameter on all dogs included in the study. To measure the agreement between observers for the EIs, EId, and EIm, the intra-class correlation coefficient was calculated along with its 95% confidence interval. This was done globally and by group, between each pair of observers, and among the four observers. Results were considered significant at the 5% significance level (*P* < 0.05). Receiver operator characteristic curve analyses were performed to determine optimal cut-off values of EId, EIs and EIm to detect moderate and severe PAH. A gray-zone approach was also applied to establish cutoff values with 90% sensitivity and specificity for EId, EIs, and EIm in detecting moderate and severe PAH. Results are presented as means and standard deviations (SD) for normally distributed data, medians and quartiles for non-normally distributed data. Comparisons between groups were done by a Chi-square test for qualitative parameters and with ANOVA for quantitative parameters with the Scheffe *post-hoc* test. All statistical analyses were carried out using SAS version 9.4 (SAS Institute, Cary, NC, USA). Statistical significance was set at *P* < 0.05.

## Results

### Patient population

Cineloops of 145 dogs were available for review and included in the study: 27 dogs without underlying heart disease (normal echocardiogram - control group), 30 dogs with stage B1 MMVD without PAH, and 88 patients with PCPH. The population demographic data is shown in Table 1. In the control group, the most common breeds included the Boxer (3), Chihuahua (2), and Vizsla (2). The median age and weight were 6.0 years (0.25–12) and 23.8 kg (2.0–48.1). The most common breeds with stage B1 MMVD included King Charles Cavalier Spaniel (6), Shih tzu (3), and Dachshund (2), Border collie (2), Australian shepherd (2), and Maltese (2). The median age and weight were 10 years (3.0–18.0) and 9.85 kg (3.3–43.6). The most common breeds with PCPH were Chihuahua (16), Shih tzu (11), Jack Russell Terrier (6), Labrador Retriever (4), Toy Poodle (3), Yorkshire Terrier (3), French Bulldog (3), Pug (3), and mixed breed dogs (6). The median age and weight were 12 years (0.4–18) and 7.2 kg (2.3–37). Dogs in the control group were significantly younger and heavier than dogs in the other groups (PAH and MMVD) (*P* < 0.001). PAH was categorized as mild in 19.3% (17/88), moderate in 35.2% (31/88), and severe in 45.5% (40/88) of the PAH group.

**Table 1:** Represented demographic data for the dogs in the control group, Pulmonary hypertension (PAH) and Myxomatous mitral valve disease group (MMVD).

	**Control**	**PAH**			**MMVD**
		**Mild**	**Moderate**	**Severe**	
Number of dogs	27/145	17/145	33/145	38/145	30/145
Age [median (range)]	6.0 (0.25–12.0)	11.5 (0.4–15)	12 (4–15.1)	11.6 (2–18)	10.0 (6.0–18.0)
Weight [median (range)]	23.8 (2.0–48.1)	9 (3.3–40)	6.5 (2.3–30.5)	7.3 (2.6- 37)	9.85 (3.3–44.0)
Male intact	6	3	5	4	1
Male neutered	14	9	14	15	9
Female intact	3	0	7	9	5
Female neutered	4	5	7	10	15

Data are expressed as median and range.

### Inter-operator agreement

**Figure 2:**
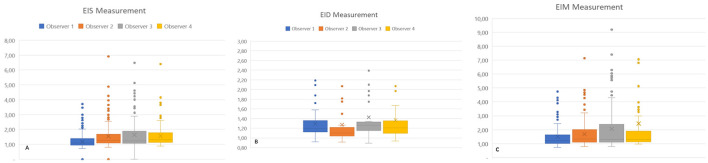
Eccentricity index measurements of individual observers for all groups. EIs: left ventricular eccentricity index measured at end-systole **(A)**. EId: left ventricular eccentricity index measured at end-diastole **(B)**; EIm: left ventricular eccentricity index measured at maximal septal flattening **(C)**.

Eccentricity indexes showed consistent agreement between each observer and every other observer in pairs (Figure 2), as well as among all observers for EIs, EId, and EIm, for both the entire population and the PAH group specifically. The median (Q1–Q3) ICC was 0.768 (0.642–0.856) for the whole population and 0.737 (0.621–0.852) for the PAH group. There was a low ICC for all observers in the MMVD group for EIs 0.304 (0.159–0.533), EId 0.530 (0.419–0.711), and EIm 0.281 (0.138–0.504).

### Eccentricity indexes in different groups

**Figure 3:**
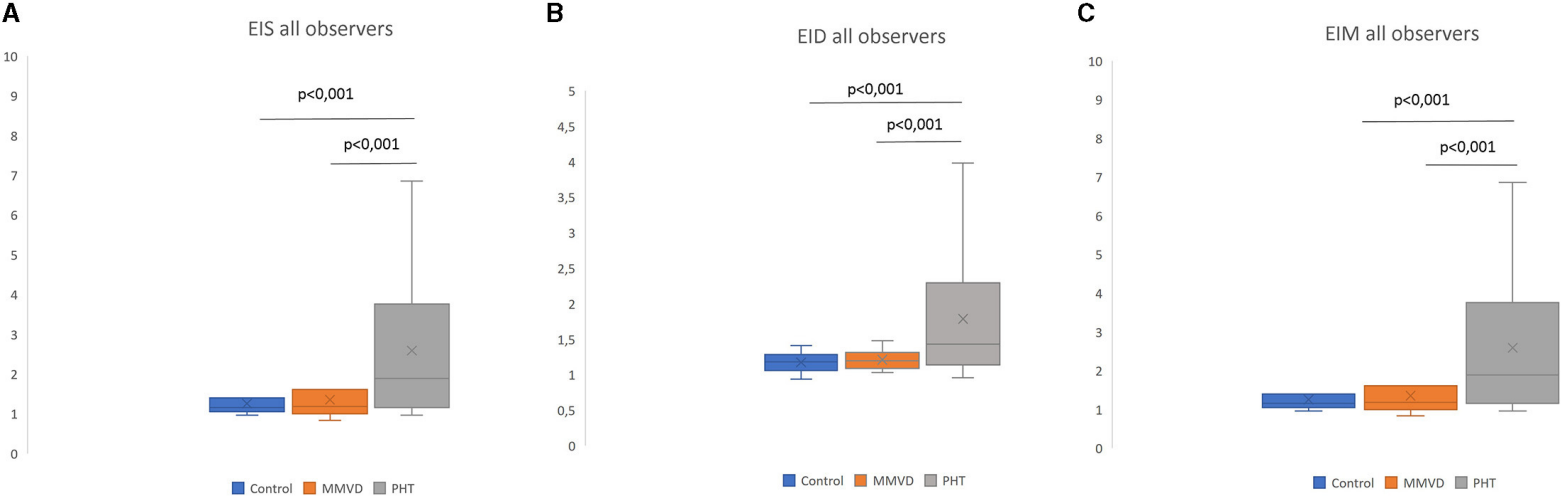
Eccentricity index among different groups (average of all observers). EIs: left ventricular eccentricity index measured at end-systole **(A)**. EId: left ventricular eccentricity index measured at end-diastole **(B)**; EIm: left ventricular eccentricity index measured at maximal septal flattening **(C)**.

EIs, EIm, and EId values were significantly higher (*p*-value < 0.0001) in the PAH group for EIs (1.17 ± 0.19), EIm (2.13 ± 1.39), and EId (1.52 ± 0.49) compared to the MMVD [EIs (1.19 ± 0.24), EIm (1.22 ± 0.31), and EId (1.20 ± 0.12)] and control group [EIs (1.17 ± 0.19), EIm (1.18 ± 0.20), and EId (1.18 ± 0.11)] (Figure 3). Patients with moderate to severe PAH had significantly higher (*p*-value 0.0014) EIs, EIm, and EId compared to dogs with mild or absent PAH (Figure 3).

### Receiver operating curves

**Figure 4:**
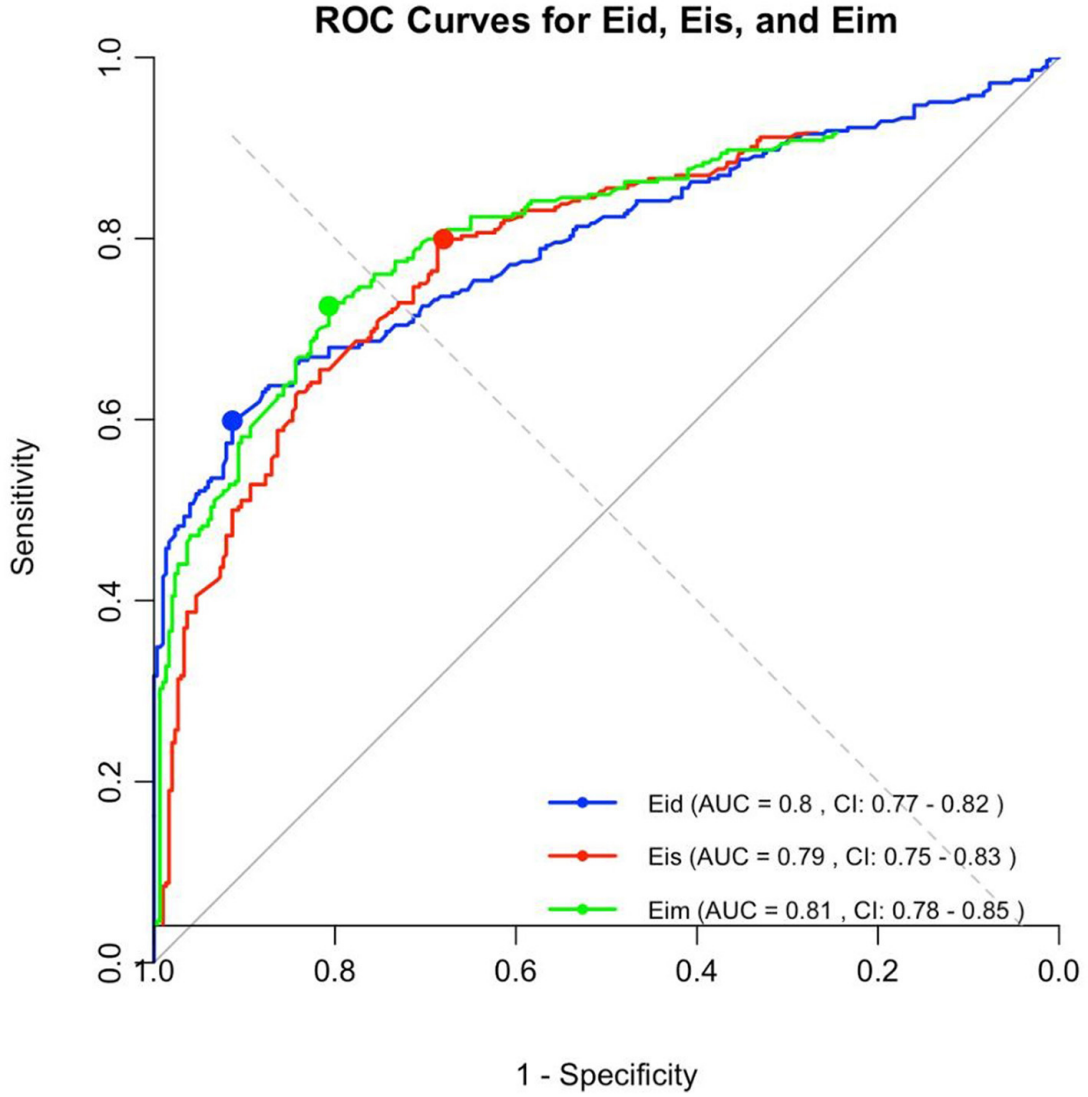
The area under the receiving operating characteristic (ROC) curve for the EIs, EId, and EIm. Optimal cutoff values of 1.28 for EIs, 1.40 for EId, and 1.34 for EIm discriminating none/mild to moderate/severe PAH, with a sensitivity of 77.3% for EIs, 65.2% for EId, and 77.3% for EIm, and a specificity of 72.7% for EIs, 100% for EId, and 77.3% for EIm.

**Figure 5:**
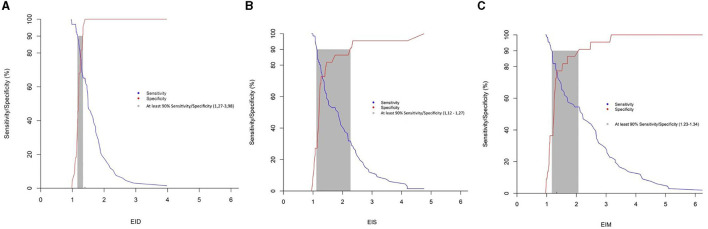
Gray zone approach for the eccentricity index **(A)** Eis; **(B)** Eid; **(C)** Eid, the blue line represents sensitivity, whilst the red line represents specificity. The gray zone represents the zone in which sensitivity and specificity are under 90%.

All EI were significantly correlated with the presence of PAH. Receiver operating curves showed an AUC of 0.738 for Eis, 0.834 for Eid, and 0.766 for EIm. The Youden index values for EIs (1.28), EId (1.40), and EIm (1.34) discriminated moderate-to-severe pulmonary arterial hypertension (PAH) from mild or no PAH, with sensitivity and specificity of 77.3 and 72.7%; 65.2 and 100%; and 77.3 and 77.3% for EIs, EId, and EIm, respectively (see Figure 4). A gray zone approach (Figure 5) indicates a 90% specificity and sensitivity for moderate-to-severe PAH of EIs, EId and EIm at values of 2.27 and 1.12; 1.32 and 1.15; and 2.1 and 1.23, respectively.

## Discussion

This study shows good ICC for EI measurement by ECC residents on prerecorded cineloops of EId, EIs, and EIm. Measures were different in moderate to severe PAH compared to normal dogs and MMVD B1.

Breed distribution was not balanced across our study population, which may have introduced a degree of variability in the measurements. Moreover, dogs in the control group were significantly younger and heavier. This is likely because a significant number of the dogs in the control group were presented for cardiac screening for breeding which probably accounts for the age difference in this group. Cardiac screening for breeding is mainly performed for diseases such as dilated cardiomyopathy, whereas screening for murmur investigation also included dogs with aortic stenosis and tricuspid dysplasia, conditions for which medium to large breed dogs are predisposed, which probably explains the heavier weight of the control group. In human medicine, similar EI cutoff values have been reported for both adult and pediatric patients. However, differences are observed in neonatal patients, suggesting that age and body weight have minimal influence on EI measurements beyond the first year of life (18). A previous veterinary cardiological study does not suggest an impact of age and weight on EI measurements (16). That said, the focus of our study was not to assess whether age and weight impact EI measurements.

The inter-observer agreement was good, with an ICC of 0.737 (0.621–0.852). The ICC for the EI was higher in one human study. The observers in these papers were however cardiologists, whereas our study was conducted by ECC residents. The higher inter-observer agreement between cardiologists could therefore be expected (9). Inter-observer agreement was good in both the PAH and control groups but was only fair in the MMVD group for all eccentricity indexes. Dogs with MMVD B1, even at a mild stage, tend to have a higher median shortening fraction compared to the reference range (19). The authors hypothesize that this increased contractility, or more kinetic profile may have impacted EI measurements. In a different study the Pulmonary Hypertension Score (PHS) appeared to be unaffected by the presence of concurrent cardiac disease when screening for PAH (13). Based on our findings, the EI should probably be interpreted with caution in dogs with concurrent cardiac disease, and the PHS may be preferrable in this setting.

Either EId, EIs, or EIm were significantly increased in dogs with PAH compared to dogs without PAH, and these values were significantly higher in dogs with moderate to severe PAH compared to dogs with mild or absent PAH. We did not aim to identify mild cases of PAH compared to the control group, as our primary objective was to support ECC clinicians in the early recognition and management of moderate to severe forms. Mild PAH cases are typically stable enough to undergo a more complete cardiac work-up at a later stage, such as the following day. Importantly, a previous study conducted by cardiologists demonstrated that the left ventricular eccentricity index was not effective in detecting mild pulmonary hypertension, supporting the idea that interventricular septal flattening becomes apparent only in more advanced disease (20).

Furthermore, EId, EIs, and EIm were significantly higher in the PAH group compared to the two control groups. EI was strongly correlated with the presence of PAH, with EId emerging as the best marker. A prior study demonstrated that EId was notably elevated in dogs with severe PAH. EId is measured when the pulmonary valve is closed, reflecting increased RV end-diastolic pressure secondary to RV failure suggesting a more pronounced impairment of right ventricular function with significant diastolic dysfunction (16).

The gray zone approach identified a cut-off of 2.27, 2.1, and 1.27 for EIs, EIm and EId, respectively, to diagnose moderate to severe PAH with 90% specificity. This cut-off may be of clinical relevance in the absence of further cardiological work-up, allowing to commence PAH therapy. Values inferior to 1.12, 1.23, and 1.27 for EIs, EIm and EId, respectively, allow the clinician to rule out moderate to severe PAH with 90% sensitivity suggesting to withhold therapy until further cardiological workup is available. The use of a gray zone approach based on 90% sensitivity and specificity thresholds has been previously used in human studies to define clinically useful diagnostic cut-offs with acceptable levels of uncertainty (15).

The Youden index identified cut-off values of 1.28, 1.40, and 1.34 for EIs, EIm and EId, respectively, to distinguish moderate-to-severe PAH from mild or no PAH. This cut-off value is comparable to those reported in a previous study conducted by cardiologists and to cut-off used in human infant to diagnose PAH (20, 21).

The proposed EI cut-offs may prove to be clinically useful for ECC clinicians in identifying patients with moderate to severe PAH. However, these cut-offs have been identified on a population without significant concurrent left heart disease, and image acquisition was performed by a cardiologist, and therefore this information should be interpreted cautiously. That said, based on our data, EI appears to show promise in an emergency setting to identify patients with moderate to severe PAH. Especially EIm may represent benefits over EIs and EId in an emergency setting as it does not require simultaneous ECG-recordings.

Our study has several limitations. In this retrospective study, some dogs had received loop diuretics or phosphodiesterase-5 inhibitor prior to echocardiography. These treatments may have influenced measurements. Studies investigating the effect of sildenafil on tricuspid regurgitation (TR) and right heart parameters have been met with variable results (3, 4, 21–23, 25). Furthermore, the severity of PAH was assessed indirectly. Doppler echocardiography-derived estimates of PAP were employed, a common procedure in a clinical setting, despite known discrepancies between non-invasive estimates and invasive measurements. Prior research indicates that while Doppler-derived TRPG can correlate with invasive PAP measurements, the correlation is not perfect (9, 24, 26). The reliance on Doppler-derived TRPG or pulmonic gradients by cardiologists for diagnosing PAH introduces additional challenges. These gradients can be influenced by several factors such as beam alignment, operator skill, sedation, body positioning, and patient respiratory patterns. Specifically, in cases with significant regurgitation, high right atrial pressure, or right ventricular systolic failure, TRPG may not accurately reflect systolic PAP. This study included instances where discrepancies between TR/pulmonic gradients and clinical indicators of right heart function were observed. To enhance diagnostic accuracy for PAH, cardiologists incorporated a comprehensive evaluation of right heart parameters alongside TR/pulmonic gradients, aligning with previously established guidelines. The population included in this study was recruited through the cardiology service, which may differ from the population seen in an emergency setting. To the authors' knowledge, there are no studies in either human or veterinary literature specifically comparing right heart echocardiographic changes between patients presenting to cardiology vs. emergency services. Therefore, whether the findings from our study can be fully extrapolated to emergency populations remains uncertain.

The study population consisted of dogs without left heart remodeling or other right-sided cardiac conditions. Furthermore, some of these cases were recruited through the cardiology service, which may not reflect the spectrum of patients typically encountered in an emergency or critical care setting. Whether ECC clinicians can reliably detect PCPH in the presence of concurrent significant left heart disease or reliably distinguish PCPH from other causes of right-sided heart disease remains an open question. Further work would be needed to assess diagnostic performance in these more complex scenarios.

In addition, the cut-off values proposed for EIs, EId, and EIm were derived from the current dataset and have not yet been validated in an independent population. External validation in a separate cohort will be necessary to confirm their generalizability and clinical applicability.

Furthermore, suboptimal angulation may influence EI measurements. However, in this study, all measurements were based on pre-recorded cineloops obtained by cardiologists. In a prior study on EI conducted by cardiologists, inter-observer variability remained within acceptable limits (7–11%), suggesting that angulation had a limited impact on our results (20).

Additionally, we acknowledge that not all ECC clinicians may be able to obtain a simultaneous ECG tracing during focused cardiac POCUS examinations. As such, the added value of EIm findings may be particularly relevant in situations where ECG monitoring is not readily available or cannot be integrated in real time.

Intra-observer agreement was not assessed in our study, which represents another limitation of our results. However, in a recent study conducted by cardiologists, Intra-observer agreement was evaluated and demonstrated good to excellent repeatability (16). In this study repeatability implied obtaining the image and measuring EI. We do acknowledge future studies in which non-cardiologists performing the measurements should assess intra-observer repeatability.

Finally, echocardiographic cineloops were acquired by board-certified cardiologists or cardiology residents on a dedicated ultrasound device. The recording of high-quality images thanks to well-trained cardiologists on superior devices may have positively impacted inter-rater agreement. Our study did not assess ability of ECC residents to obtain these cineloops, but their capacity to measure EI on these cineloops. Prospective studies in which non-cardiologists record and interpret the images are currently ongoing.

## Conclusion

This study showed good inter-observer agreement of EIs, EIm, and EId measurement by ECC residents on prerecorded loops. EI allowed good identification of dogs with moderate to severe PAH by ECC residents.

## Data Availability

The raw data supporting the conclusions of this article will be made available by the authors, without undue reservation.

## Footnotes

^1^GE vivid E95 equipped with 2.2–3.5 and 5.5–7.5 and 1.4–4.6 and 2.4–8.0 MHz phased-array transducers and a Phillips Epiq 7 equipped with 12-4, 8-3, and 5-1 MHz traducers.
